# Complete genome sequence of *Anaerococcus* sp. strain AH8042_DFU013_CI05 isolated from a diabetes-related foot ulcer

**DOI:** 10.1128/mra.01326-24

**Published:** 2025-03-25

**Authors:** Nan Hao, Sarah Vreugde, Robert Fitridge, Keith E. Shearwin

**Affiliations:** 1 Department of Molecular and Biomedical Science, School of Biological Sciences, The University of Adelaide 1066, Adelaide, South Australia, Australia; 2 Department of Surgery-Otolaryngology Head and Neck Surgery, Adelaide Medical School, The University of Adelaide 110466, Adelaide, South Australia, Australia; 3 Basil Hetzel Institute for Translational Health Research, Central Adelaide Local Health Network 375072, Adelaide, South Australia, Australia; 4 Discipline of Surgical Specialties, Adelaide Medical School, The University of Adelaide 1066, Adelaide, South Australia, Australia; 5 Department of Vascular and Endovascular Surgery, Royal Adelaide Hospital 1062, Adelaide, South Australia, Australia; Loyola University Chicago, Chicago, Illinois, USA

**Keywords:** anaerococcus, foot ulcer

## Abstract

This study presents the complete genome sequence of *Anaerococcus sp*. strain AH8042_DFU013_CI05, isolated from a diabetes-related foot ulcer at The Queen Elizabeth Hospital in Adelaide, Australia. The genome comprises a 1,752,963 bp circular chromosome and a 14,073 bp plasmid, with G+C contents of 32% and 29%, respectively.

## ANNOUNCEMENT

The genus *Anaerococcus,* first proposed in 2001 with *Anaerococcus prevotii* as its type species (1), comprises 15 validly described species of gram-positive, strictly anaerobic bacteria listed in the LPSN (List of Prokaryotic Names with Standing in Nomenclature) (2). These species are frequently isolated from clinical specimens, including abscesses and infections at various body sites (3). Notably, *Anaerococcus* species are highly prevalent in diabetes-related foot ulcers (4–6), underscoring their potential role in the pathogenesis of these infections and highlighting the importance of sequencing to elucidate their contribution to wound microbiota.

A swab sample was collected from a diabetes-related ulcer on the left foot of a male patient at the Multi-disciplinary Foot Clinic, The Queen Elizabeth Hospital, Adelaide. Ethics approval and written informed consent were obtained prior to sample collection. After cleaning and debriding the wound, a swab was taken using the Levine technique (7), placed into Sigma Transwab Liquid Amies solution, and transferred to an anaerobic workstation. The sample was plated on tryptic soy agar supplemented with 5% defibrinated sheep blood and incubated anaerobically at 37°C for 3 days. Species identification using matrix-assisted laser desorption ionization-time of flight (MALDI-TOF) mass spectrometry revealed six known bacterial species and one unidentified species, designated AH8042_DFU013_CI05.

Strain AH8042_DFU013_CI05 was propagated anaerobically in the Brain Heart Infusion medium with 0.2% Tween 80 for 3 days at 37°C and harvested by centrifugation. Genomic DNA was extracted using the DNeasy Blood & Tissue Kit (Qiagen). A Nanopore sequencing library was prepared with the SQK-RBK114.96 Rapid Barcoding Kit and sequenced on a GridION sequencer using an R10.4.1 flow cell. Reads were base-called and demultiplexed with Guppy v4.2.0 in super-accurate (SUP) mode.

*De novo* assembly was performed with Flye v2.9.2-b1786 (8) following the Dragonflye v1.0.5 pipeline (Robert A. Petit III, Wyoming Public Health Laboratory). Assembly (Table 1) revealed a single circular chromosome (1,752,963 bp) and a plasmid (14,073 bp). The genome was annotated with Bakta v1.10.1 (db5.1.0) (9), identifying the features listed in Table 1. PHASTER (10) further predicted the presence of one intact prophage. Default parameters were used for assembly and annotation.

**TABLE 1:** Summary of sequence data

Parameters	Value
Number of contigs	2
Number of reads	63,268
N_50_	8,897 bp
Genome sizes: chromosome; plasmid	1,752,963 bp; 14,073 bp
Coverage: chromosome; plasmid	142 ×; 410 ×
G + C content: chromosome; plasmid	32%; 29%
Number of coding sequences (CDS)	1,749
Number of tRNA genes	47
Number of tmRNA genes	1
Number of rRNA genes	12
Number of ncRNA genes	5
Number of CRISPR loci	1
Accession numbers: chromosome; plasmid	CP175954; CP175955

**Fig 1:**
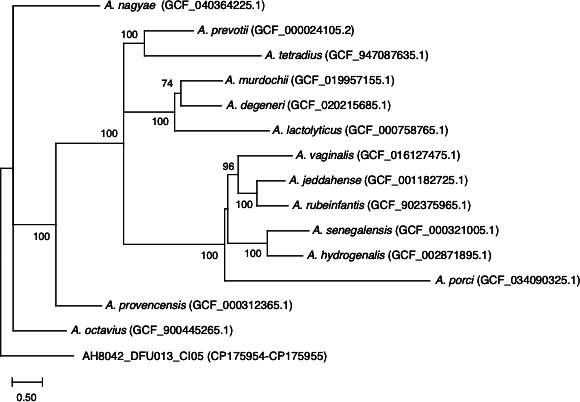
Phylogenetic tree of *Anaerococcus* species constructed using kSNP4.1 (13) with an optimal k-mer size of 19 and 6,824 SNPs, employing a parsimony algorithm to minimize evolutionary steps. The tree was visualized in MEGA v11.0.13 (14), with genome accession numbers indicated in parentheses. Bootstrap values (100 replicates) are shown at nodes.

Ribosomal Multilocus Sequence Typing (rMLST) (11) identified AH8042_DFU013_CI05 as belonging to the genus *Anaerococcus* but failed to assign it to a specific species. Average nucleotide identity (ANI) analysis with FastANI v1.34 (12) revealed that the isolate shares 80.18% identity with the type strain *Anaerococcus prevotii* DSM 20548 (NCBI: ASM2410v1). A phylogenetic tree constructed with 14 *Anaerococcus* species from the LPSN indicated that AH8042_DFU013_CI05 is evolutionarily distinct from other known *Anaerococcus* species (Fig. 1).

## Data Availability

The raw reads for genome sequencing have been deposited in the NCBI Sequence Read Archive under accession numbers SRR31626410. The AH8042_DFU013_CI05 genome sequence was deposited in GenBank under accession numbers CP175954 and CP175955 (BioProject/BioSample PRJNA1194632/SAMN45194444).
